# Hepatoprotective and Antioxidant Activities of Oil from Baru Almonds (*Dipteryx alata* Vog.) in a Preclinical Model of Lipotoxicity and Dyslipidemia

**DOI:** 10.1155/2018/8376081

**Published:** 2018-10-02

**Authors:** Mayara Áthina Reis, Rômulo Dias Novaes, Sueli Regina Baggio, André Luiz Machado Viana, Bruno Cesar Correa Salles, Stella Maris da Silveira Duarte, Maria Rita Rodrigues, Fernanda Borges de Araújo Paula

**Affiliations:** ^1^Departamento de Análises Clínicas e Toxicológicas, Faculdade de Ciências Farmacêuticas, Universidade Federal de Alfenas (UNIFAL-MG), Alfenas, Minas Gerais, Brazil; ^2^Departamento de Biologia Estrutural, Instituto de Ciências Biomédicas, Universidade Federal de Alfenas (UNIFAL-MG), Alfenas, Minas Gerais, Brazil; ^3^Centro de Ciência e Qualidade de Alimentos, Instituto de Tecnologia de Alimentos (ITAL), Campinas, São Paulo, Brazil

## Abstract

The oil obtained from baru (*Dipteryx alata* Vog.) almonds exhibits high energy value and is reported in popular medicine for the treatment of rheumatic diseases and reproductive disturbances. Although baru oil is used in domestic cuisine, the chemical characterization of this oil and its effects on lipid metabolism are still poorly understood. Therefore, this study evaluated the fatty acid (FA) profile and the effects of baru oil on liver and aorta in a murine model of dyslipidemia. The chromatographic profile of baru oil showed high levels of unsaturated FAs, especially oleic acid. Saturated FAs, such as palmitic and lignoceric acids, were found in lower amounts. Hypercholesterolemia was induced in male Wistar rats by daily administration of a lipid emulsion by gavage for 15 weeks. Biochemical and histopathological analysis were performed on serum, aorta, and liver. The results demonstrated that animals developed marked hypercholesterolemia, liver steatosis, and increased lipid peroxidation in the aorta. Treatment with baru oil attenuated lipid peroxidation and drastically reduced liver damage, especially ballooning degeneration and steatosis. By restricting vascular and hepatic injury, this oil showed potential applicability as a functional food, reinforcing its use in popular medicine and domestic cuisine.

## 1. Introduction

It is known that lipotoxocity from high levels of free fatty acids, free cholesterol, and other lipid metabolites can result in cell death, mitochondrial dysfunction, endoplasmic reticulum stress, ceramide accumulation, increased exposure to reactive oxygen species (ROS), and increased lipid peroxidation in various organs and tissues [1–3]. Both direct toxic effects and those secondary to lipotoxicity may contribute to the development of chronic diseases related to dyslipidemia, such as nonalcoholic fatty liver disease (NAFLD) [2, 4]. NAFLD may progress to nonalcoholic steatohepatitis, fibrosis, and cirrhosis with increased risk of hepatocellular carcinoma. Moreover, NAFLD confers an increased risk of cardiovascular disease (CVD), occupying a prominent position in the world scenario in view of its prevalence and the high morbidity and mortality associated with it, as well as the high cost to public services and reduced quality of life in patients with these diseases [5, 6].

Despite the rising prevalence, until now, definitive treatment for NAFLD has not yet been established. New therapeutic approaches to protecting the liver against damage induced by lipotoxicity are indispensable. [7]. Studies related to evaluation of the biological activity of edible vegetables or their bioactive compounds have attracted great attention in recent years due to their potential as functional foods and nutraceuticals [8]. Therefore, the application of functional foods in the prevention or treatment of chronic diseases has been a new trend in several countries, including Brazil [9]. Because of their efficacy and low cost, many edible vegetables are used as alternative or complementary treatment for several diseases, especially in popular medicine [8, 9]. However, not all have had their biological activity evaluated in scientific studies [9].

Belonging to the family Leguminosae Faboideae, the plant species* Dipteryx alata* Vog, popularly known as baru, is native to the Brazilian Cerrado biome and constitutes a source of income for the regional population by providing wood, fruits, and almonds [10]. The almonds and pulp obtained from the baru fruits are intended for human consumption and are used in different candy recipes and liqueurs produced in regional cuisine [11, 12]. Oil has been obtained from baru almonds with high energy value which is used in domestic cuisine [13]. Furthermore, it has been reported that baru oil may have potential therapeutic properties, especially considering its use in popular medicine for the treatment of rheumatic diseases and reproductive disturbances [10].

Some authors have demonstrated the presence of monounsaturated and polyunsaturated fatty acids, such as oleic, linoleic, palmitic, lignoceric, stearic, behenic, gadoleic, and araquidic acids in baru oil [10, 12, 14, 15]. The presence of these fatty acids (FA) in baru oil may have important implications for healthcare, since the amount and type of ingested FA can modulate pathways associated with lipotoxicity, producing beneficial or harmful effects on health [3, 16]. Consumption of baru almonds has been reported to decrease oxidative stress in the liver and spleen of rats [17]. Bento et al. observed improvement in the serum lipid profile of subjects with moderate dylsipidemia [18] Previous in vitro and in vivo studies indicated that these almonds contained secondary metabolites with high antioxidant potential, especially phenolic compounds, catechin, ferulic acid and vitamin E [15, 17, 19, 20].

However, to the best of our knowledge, there is little information available concerning the in vivo effects of baru oil on the health. Therefore, the objective of this study was to evaluate the profile of fatty acids present in oil from baru almonds and to investigate their effects on liver morphology and function, serum markers of cardiovascular risk and lipid peroxidation in the aorta using a murine model of hyperlipidaemia.

## 2. Materials and Methods

### 2.1. Plant Material and Oil Extraction

The baru almonds were collected in a cerrado biome and purchased from local producers in the Januária region, Minas Gerais, Brazil. The almonds were selected and roasted at 150°C for 45 minutes in an oven with air circulation, as previously described [15]. The almond oil was extracted by mechanical pressing at room temperature (28°C) with initial and final pressures of 3 and 12 tons, respectively [21]. The oil was then filtered and stored in amber vials at 8°C.

### 2.2. Evaluation of Fatty Acids Profile

The fatty acid composition was determined by gas phase chromatography coupled to mass spectrometry (GC-MS; QP-2010 Plus Shimadzu® Corporation, Kyoto, Japan). The preparation of fatty acid methyl esters was performed according to a previously described method by Hartman & Lago [22] and the chromatographic conditions were as reported by Firestone [23] and Horwitz [24].

### 2.3. Animals

Male Wistar rats (n = 40) weighing 400 ± 25 g were housed in a controlled environment with a temperature of 22 ± 2°C, 40–60% humidity and 12/12-h light-dark cycle. Animals received water and commercial food ad libitum. The study was approved by the Ethics Committee in Use of Animals of the University (CEUA/UNIFAL-MG, protocol no 06/2016). After 5 days of acclimatisation, the animals were randomly assigned to four groups and treated as follows: control group (C) received drinking water; baru oil group (BO) received baru oil; hypercholesterolaemic group (HC) received lipid emulsion; hypercholesterolaemic-baru oil group (HC-BO) received lipid emulsion and baru oil. The baru oil and lipid emulsion were administered by gavage at a dose of 1 g/kg/day and 10 mL/kg, respectively. It was based in studies of Sekhon-Loodu et al. [25] and Li et al. [26]. All treatments were administered daily for 15 weeks. Animal body weight was recorded on a weekly basis. Daily feed and water intake was checked three times a week during the 15-week treatment period. At the end of treatment, the animals were subjected to fasting for 12 h and then anaesthetized. Blood was collected by puncture from the abdominal aorta to obtain serum. Animals were then euthanized for collection of the liver and the aorta. The liver was removed and stored in 10% formaldehyde. The aorta was homogenized in 0.1M phosphate buffered saline containing protease inhibitors and stored at 4°C.

### 2.4. Lipid Profile and Hepatic Function

The lipid profile of serum samples was assessed by means of determinations of total cholesterol, triacylglycerols and HDL cholesterol using enzymatic colorimetric methods. The concentration of non-HDL cholesterol was calculated as the difference between total cholesterol and HDL cholesterol levels, according to Xavier et al., [27] and reflects the pool of atherogenic lipoproteins in the bloodstream. Hepatic function was evaluated by the quantification of serum albumin using the bromocresol green method and activity of the enzymes aspartate aminotransferase (AST), alanine aminotransferase (ALT) and alkaline phosphatise (ALP) by kinetic methods. All analyses were performed using the LabMax Pleno automatic system with commercial kits (Labtest, Lagoa Santa, Minas Gerais, Brazil).

### 2.5. Lipid Peroxidation Assay

Lipid peroxidation was determined in serum and homogenized aorta by quantification of malonaldehyde (MDA) using fluorimetry, according to Brown & Kelly [28]. The MDA concentration was calculated from a calibration curve using tetraethoxypropane.

### 2.6. Morphological Analysis

After euthanasia, the liver was submitted to macroscopic examination, fixed in 10% formaldehyde for 48 h and then prepared for microscopic examination, according to Freitas et al. [19] and Novaes et al. [29]. For each animal, random histological images were obtained at 400× magnification using a bright field microscope coupled with a digital camera. Digital images were used for histopathological examination of ballooning degeneration, steatosis, inflammation, and necrosis. The morphological reorganisation of the liver tissue was evaluated by the stereological method. Image Pro-Plus 4.5 software was used (Media Cybernetics, Silver Spring, MD, USA) in all morphological analyses [29].

### 2.7. Statistical Analysis

Results were expressed as mean values and standard error of mean (mean + SEM). Data distribution was investigated using the Kolmogorov-Smirnov test. Parametric data were submitted to unifactorial one-way analysis of variance (ANOVA) followed by the Tukey post hoc test for multiple comparisons. Nonparametric data were compared by the Kruskal-Wallis test. Statistical significance was established at p < 0.05.

## 3. Results

**Figure 1:**
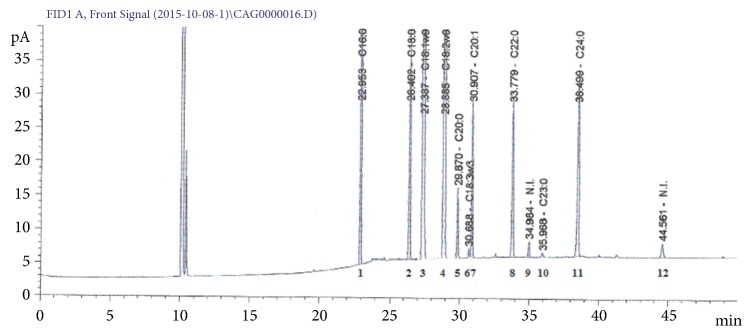
Chromatogram of baru oil analysis. C16:0: palmitic acid, C18:0: stearic acid, C18:1w9: oleic acid, C18:2w6: linoleic acid, C20:0: arachidic acid, C18:3w3: linolenic acid, C20:1: cis-11-eicosenoic acid, C22:0: behenic acid, C23:0: tricosanoic acid, C24:0: lignoceric acid, and N.I.: not identified.

Approximately 270 mL oil was obtained per 1000 g baru roasted almonds, with an estimated average yield of 30% (w/v). The chromatographic profile of baru oil is shown in Figure 1.

Monounsaturated fatty acids (MUFAs) were predominant, followed by polyunsaturated fatty acids (PUFAs). Among the MUFAs, oleic acid was predominant, while, among the PUFAs, linoleic acid was most abundant. Saturated fatty acids (SFAs) were observed in smaller amounts, such as palmitic acid, followed by lignoceric, stearic, behenic, and arachidic acids (Table 1).

In the in vivo study, there was no difference in weight gain between the different experimental groups, but there was a reduction in food and water intake in groups HC and HC-BO compared to animals in groups C and BO (Table 2).

The lipid emulsion was effective in inducing dyslipidaemia, demonstrated by increased levels of TC and non-HDL cholesterol and reduced HDL cholesterol serum levels in groups HC and HC-BO compared to groups C and BO. Treatment with baru oil did not modify the serum lipid profile. No significant difference was observed in glycaemia among the groups. The activities of AST, ALT, and ALP, as well as serum levels of albumin, were similar in all groups (Table 3).

**Figure 2:**
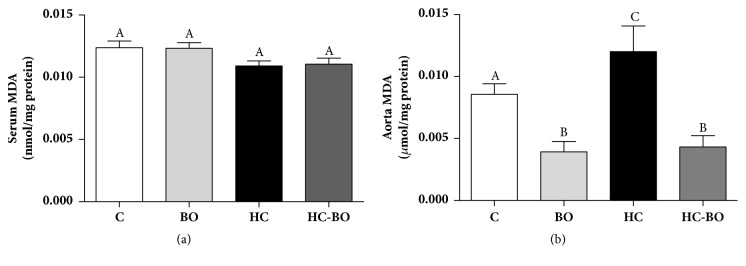
Malondialdehyde (MDA) levels in serum (a) and aorta artery (b) of rats treated with water (C), baru oil (BO), hypercholesterolemic (HC), and hypercholesterolemic + baru oil (HC-BO) for 15 weeks. Data are presented as the means ± SEM. Values with different superscript letters in the same row are significantly different (p < 0.05), ANOVA followed by Tukey's test.

MDA serum levels was similar in both groups (Figure 2(a)). In contrast, there was a marked increase in MDA levels in the aorta in group HC compared to the other groups. This parameter was significantly reduced in both groups treated with baru oil compared to the controls (Figure 2(b)).

**Figure 3:**
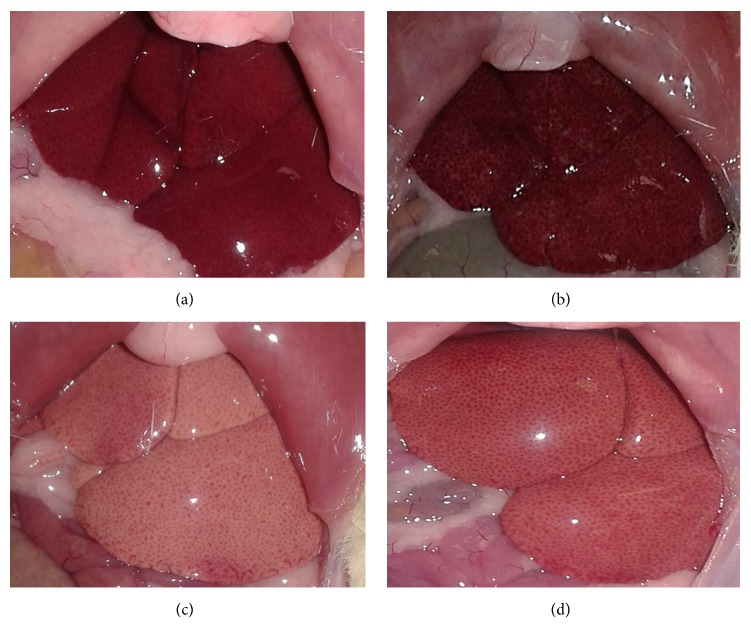
Macroscopic images of liver from rats treated with treated with water (a), baru oil (b) and hypercholesterolemic rats (c) and hypercholesterolemic rats treated with baru oil (d) for 15 weeks. In the animals treated with high-fat emulsion ((c) and (d)), note the liver discoloration and granular pattern indicative of hypovascularization and congestion of intrahepatic vessels. This pattern was not observed in the animals treated with baru oil (b).

In animals treated with lipid emulsion, especially in group HC, macroscopic changes in liver tissue were observed, such as increased size, opacification, and granular aspect (Figure 3).

**Figure 4:**
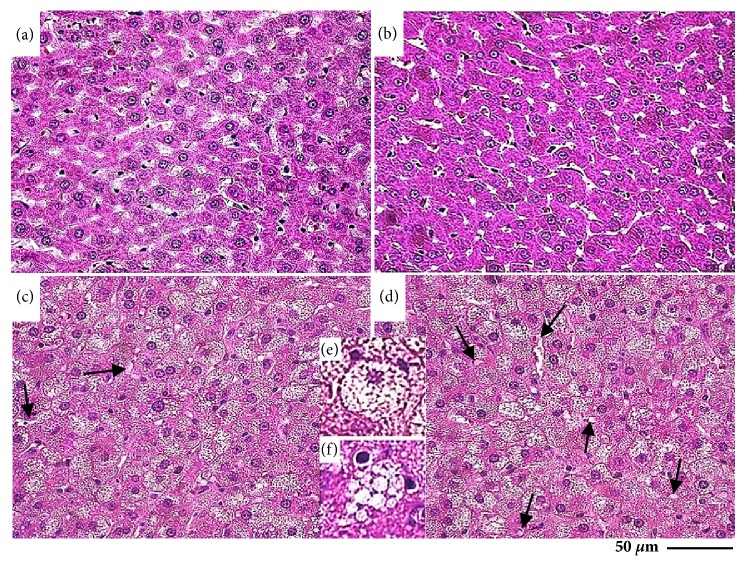
Representative photomicrographs of the liver tissue from rats treated with water (a), baru oil (b) and hypercholesterolemic rats (c) and hypercholesterolemic rats treated with baru oil (d) for 15 weeks. The images (e) and (f) indicate ballooning degeneration and steatosis, respectively. The arrows point sinusoid capillaries.

The administration of lipid emulsion induced a drastic morphological remodeling in liver tissue in HC animals, which was attenuated by the treatment with baru oil (Figure 4).

**Figure 5:**
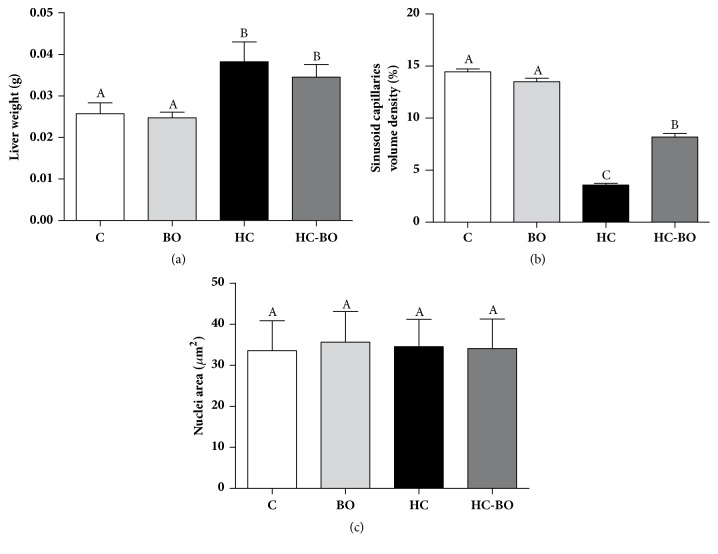
Liver weight (a), vascular distribution (b) and hepatocyte nucleus size (c) in rats treated with water (C), baru oil (BO), hypercholesterolemic (HC), and hypercholesterolemic + baru oil (HC-BO) for 15 weeks. Data are presented as the means ± SEM. Values with different superscript letters (A, B, C) in the same row are significantly different (p < 0.05), Kruskal-Wallis test.

Liver weight was increased in groups HC and HC-BO compared to groups C and BO (Figure 5(a)). The volume density of capillary sinusoids was reduced in group HC-BO compared to groups C and BO (Figure 5(b)). The size of hepatocyte nuclei was similar in both groups (Figure 5(c)).

**Figure 6:**
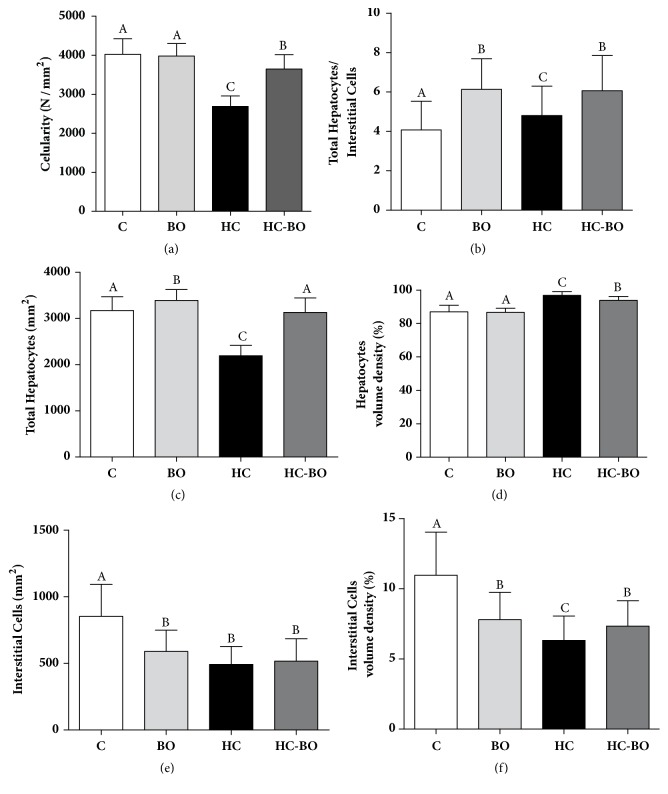
Cellularity ((a) and (b)), relative and absolute distribution of hepatocytes ((c) and (d)) and interstitial cells ((e) and (f)) and in the liver tissue from rats treated with water (C), baru oil (BO), hypercholesterolemic (HC), and hypercholesterolemic + baru oil (HC-BO) for 15 weeks. Data are presented as the means ± SEM. Values with different superscript letters (A, B, C) in the same row are significantly different (p < 0.05), Kruskal-Wallis test.

A significant reduction in relative and absolute distribution in interstitial cells and total cellularity was observed, as well as a significant reduction in the number of hepatocytes per unit of area examined in group HC compared to the other groups (Figures 6(a), 6(c), 6(d)–6(f)). The TH/IC index was higher in the groups BO, HC, and HC-BO compared to the group C. This parameter was reduced in the group HC compared to the groups BO and HC-BO (Figure 6(b)).

**Figure 7:**
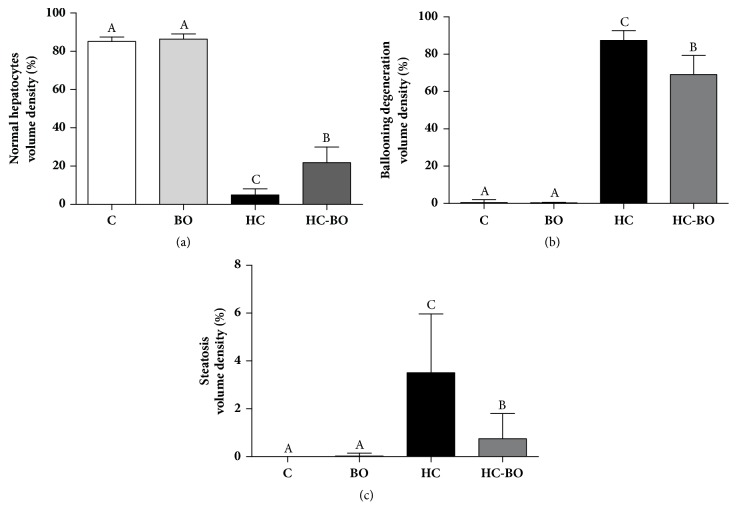
Distribution of normal and degenerated hepatocytes in rats treated with water (C), baru oil (BO), and hypercholesterolemic (HC) and hypercholesterolemic + baru oil (HC-BO) for 15 weeks. Data are presented as the means ± SEM. Values with different superscript letters (A, B, C) in the same row are significantly different (p < 0.05), Kruskal-Wallis test.

The number of normal hepatocytes was reduced in the animals receiving lipid emulsion, especially group HC which showed a reduced value compared to group HC-BO (Figure 7(a)). Conversely, there were a large number of degenerated hepatocytes (ballooning and steatosis) in both groups HC and HC-BO compared to groups C and BO. Both types of degeneration were attenuated in group HC-BO compared to HC (Figures 7(b) and 7(c)).

## 4. Discussion

Among the various methods available for the extraction of vegetable oils, extraction by pressing is one of the most common methods, followed by extraction with solvents. Our results showed that the yield of baru oil from mechanical pressing, employed in this study, was similar to solvent extraction [12]. The oil obtained showed a FA profile similar to that observed by other authors [15, 30–32] with the exception of alpha-linolenic acid (C 18:3, *ω*-3 *α*). The *ω*-3*α* content found in our study was lower than that previously reported [15]. This difference is expected since factors, such as genetic variety, climate, time of year, soil type, storage process, and method of extraction and analysis can directly affect the chemical composition of oil from in several plant species [33, 34]. Due to the FA profile and predominance of unsaturated lipids, baru oil seems to be adequate for the human consumption, corroborating the use of this oil in domestic cuisine [30].

Treatment with lipid emulsion was effective in inducing dyslipidaemia, although was not accompanied by weight gain. Apparently, this finding was associated with lower consumption of food and water in animals treated with this emulsion. Changes in food intake are influenced by several environmental factors, including diet composition [35]. It has been shown that, due to their high energy value, hypercalorific diets lead to a reduced food intake [36] a characteristic that could partially explain the results observed in group HC and HC-BO. As this finding was not observed in group BO, it is possible that the changes in dietary intake are influenced more by the characteristics of lipid emulsion than baru oil.

As in the present study, environmental dyslipidaemia has been strongly associated with the development of chronic diseases and lipotoxicity, especially involving the liver and blood vessels [2, 3, 37]. Several treatments have been proposed to control lipid levels and reduce the risk of metabolic diseases [27, 38]. It has been shown that the combination of monounsaturated and polyunsaturated fatty acids may contribute to the reduction in TC and LDL cholesterol without necessarily reducing HDL cholesterol. This suggests an important cardioprotective activity of these compounds [30, 39]. In addition, unsaturated FAs have frequently been associated with modulation of energy metabolism, directly acting in the prevention of diseases associated with hepatic and vascular lipotoxicity [12, 40, 41].

Although baru oil contained predominantly MUFAs and PUFAs, especially oleic and linoleic acids, treatment with this oil was unable to induce significant changes in serum lipids and glycaemia. Sekhon-Loodu et al. [25] demonstrated that treatment of dyslipidaemic rats with fish oil containing 75.7% PUFAs was effective in controlling lipid metabolism, reducing serum levels of TAG and non-HDL cholesterol at the same time as increasing HDL cholesterol levels. The concentration of PUFAs in baru oil used in this study was estimated to be 28.19% (w/w), which could explain the absence of an effect of oil on the lipid profile. It is important to consider that SFAs could have the opposite effects to PUFAs on serum cholesterol levels and may increase serum levels of TC and TAG [27, 42]. Therefore, the neutral effect of baru oil on lipid metabolism may be associated not only with the relatively lower concentration of PUFAs compared to other oils, but also with its FA profile, since different lipid compositions can cause varying effects on energy metabolism [42]. Previous studies have suggested that the beneficial effect of baru oil intake may not be exclusively associated with the fatty acid profile. Thus, components such as flavonoids, terpenes, and sterols can contribute to the adjustment of redox balance and lipid metabolism [17, 20, 30, 31], with potential cardiovascular repercussions [18] that are still poorly understood and require further studies.

Considering that baru oil is rich in linoleic and linolenic acids and that these FAs had positive effects on energy metabolism, and prevention and control of lipotoxicity [2, 3, 37] glycaemia and lipid peroxidation were also investigated. Our results demonstrated that treatment with the lipid emulsion, as well as baru oil, did not influence carbohydrate metabolism. The evaluation of glycaemia becomes important, since that blood glucose levels can be associated with disorders in lipid metabolism and the occurrence of NAFLD [43], as well as cardiovascular diseases [16].

The association between hypercholesterolemia and increased lipid peroxidation has been demonstrated in several studies [26, 44, 45]. Although similar serum levels of MDA were detected in all treatment groups. MDA content was higher in the aorta from HC animals. However, this marker of lipid peroxidation was drastically reduced by treatment with baru oil in HC-BO animals. This finding is consistent with effects of dyslipidaemia on lipid peroxidation, since different organs and tissues present varying repertoires of antioxidant defences, which are responsible for the broad spectrum of resistance and susceptibility to lipid peroxidation [46]. Thus, the presence and activity of different enzymatic and nonenzymatic antioxidant systems could be linked to the varying ability of serum and the aorta to neutralize ROS and control oxidative stress [47] As the administration of baru oil drastically reduced MDA levels in the aorta of hyperlipidaemic and nonhyperlipidaemic animals, this oil could be a food product with potential application in conditions associated with vascular oxidative stress. Although the presence of compounds with antioxidant activity, such as terpenes, tocopherols and phytosterols, in almonds and baru oil has been demonstrated by some authors [15, 20, 31, 48] this issue is poorly understood, particularly in vivo and requires further investigation.

The macroscopic and microscopic pathological changes identified in the liver (discoloration and opacification, increased weight, and reduced distribution of sinusoids capillaries, hepatocytes and interstitial cells) indicated that administration of lipid emulsion presented an effective model of liver damage. From this model it was possible to establish that treatment with baru oil induced positive repercussions on liver structure, especially the attenuation of hepatocyte ballooning degeneration and steatosis. Ballooning degeneration was the main pathological manifestation attenuated by treatment with baru oil. This degenerative process is characterized by water accumulation in hepatocytes, resulting in initial hepatic lesions involve cell hypertrophy and can develop into disturbances in energy metabolism and steatosis [43]. Thus, the reduction in liver damage indicates that baru oil could be used as a potential strategy for controlling the development of NAFLD induced by environmental dyslipidaemia. Even in the presence of evident morphological liver damage, the activity of the enzymes AST, ALT, and ALP and albumin levels were similar in groups HC and HC-BO. This finding supports the hypothesis that the model used here induced initial liver injuries, although we were not able to demonstrate cell lysis or cholestasis, conditions directly associated with high serum levels of transaminases [49]. The tissue lesions that occur in advanced stages of NAFLD, such as steatohepatitis, liver fibrosis, and cirrhosis, are aggressive hepatic lesions which involve massive hepatocyte necrosis and increased circulating levels of aminotransferases [50]. Even in the initial phase of liver disease, the hepatoprotective effect of baru oil was a remarkable finding, particularly considering that nontreated conditions (i.e., dyslipidaemia) can develop into NAFLD, liver failure, and death [51, 52]. Although poorly understood, these beneficial effects could be related to the possible action of baru oil on lipid metabolism, especially considering that unsaturated fatty acids and antioxidant compounds can modulate liver metabolic pathways involved in lipotoxicity [40, 41, 53].

## 5. Conclusions

Taken together, our results indicate that baru oil has a beneficial composition of fatty acids with high biological value. Monounsaturated fatty acids were predominant in baru oil, while polyunsaturated fatty acids and saturated fatty acids were detected at low levels. By restricting vascular lipid peroxidation and hepatic morphological damage in a murine model of dyslipidaemia and lipotoxicity, baru oil showed potential applicability as a functional food.

## Figures and Tables

**Table 1:** Fatty acids profile in the oil extracted from baru roasted almonds.

Chain length	Common name	% Area	g/100 g*∗*
Saturated		20.14	19.25

C 16:0	Palmitic	6.14	5.87
C 18:0	Stearic	4.54	4.34
C 20:0	Arachidic	1.20	1.15
C 22:0	Behenic	3.33	3.18
C 23:0	Tricosanoic	0.08	0.08
C 24:0	Lignoceric	4.85	4.64

Monounsaturated		49.58	47.40

C 18:1	Oleic	46.71	44.65
C 20:1	Cis-11-eicosenoic	2.87	2.74

Polyunsaturated		29.49	28.19

C 18:2	Linolenic	29.34	28.05
C 18:3	*α*-linolenic	0.15	0.14

Not identified		0.79	0.76

TOTAL		100.0	

*∗*Content of fatty acids in 100g of baru oil.

**Table 2:** Average body weight, food and water intake of rats treated with lipids emulsion and baru oil.

Body weight (g)				Food intake (g)	Water intake (mL)
Goups	1st week	8th week	15th week	1-15th week	1-15th week

C	160.8±25.6^a^	362.3±27.9^a^	425.9±34.3^a^	101.1±3.5^a^	125.8±2.3^a^
BO	162.0±25.7^a^	350.6±30.2^a^	408.3±27.1^a^	102.2±4.2^a^	126.8±3.0^a^
HC	163.4±27.3^a^	346.4±32.7^a^	405±39.7^a^	89.4±3.2^b^	120.6±2.5^b^
HC-BO	170.2±26.6^a^	364.3±24.7^a^	413.3±23.3^a^	93.6±2.7^b^	115.2±4.2^b^

All data are reported as mean values for the indicated periods. Control (C), baru oil (BO), hypercholesterolemic (HC) and hypercholesterolemic + baru oil (HC-BO). Data are presented as the means ± SEM. Values with different superscript letters in the same row are significantly different (p < 0.05), ANOVA followed by Tukey's test.

**Table 3:** Biochemical parameters of rats treated with lipids emulsion and baru oil.

Parameters (mg/dL)	**Groups**			
	**C**	**BO**	**HC**	**HC-BO**
TC	77.9 ± 10.9^a^	77.7 ± 11.9^a^	137.4 ± 19.5^b^	129.2 ± 22.6^b^
Non-HDL-C	33.0 ± 8.5^a^	35.7± 9.4^a^	104.4 ± 17.4^b^	96.4 ± 21.0^b^
HDL cholesterol	44.9 ± 3.2^a^	42.0 ± 2.8^a^	32.8 ± 4.4^b^	31.8 ± 5.0^b^
Triacylglycerols	77.8 ± 15.4^a^	70.0 ± 20.1^a^	64.1 ± 18.5^a^	62.3 ± 21.8^a^
Glycemia	160.8 ± 14.9^a^	156.4 ± 10.2^a^	169.9 ± 16.2^a^	169.1 ± 14.8^a^
ALT (U/L)	119.7 ± 20.1^a^	120.5 ± 13.1^a^	116.2 ± 13.7^a^	112.1 ± 31.9^a^
AST (U/L)	36.2 ± 9.5^a^	35.6 ± 5.3^a^	37.6 ± 6.1^a^	37.1 ± 7.7^a^
ALP (U/L)	77.1 ± 17.2^a^	80.8 ± 14.0^a^	95.1 ± 15.6^a^	83.3 ± 10.6^a^
Albumin (g/dL)	3.04 ± 0.1^a^	2.96 ± 0.1^a^	3.09 ± 0.1^a^	2.98 ± 0.2^a^

TC, Total cholesterol; Non-HDL-C, non-HDL cholesterol; ALT, Alanine aminotransferase; AST, Aspartate aminotransferase; ALP, Alkaline phosphatase. Control (C), baru oil (BO), hypercholesterolemic (HC) and hypercholesterolemic + baru oil (HC-BO). Data are presented as the means ± SEM. Values with different superscript letters in the same row are significantly different (p < 0.05), ANOVA followed by Tukey's test.

## Data Availability

The data used to support the findings of this study are available from the corresponding author upon request.
